# KPNA2 interaction with CBX8 contributes to the development and progression of bladder cancer by mediating the PRDM1/c-FOS pathway

**DOI:** 10.1186/s12967-021-02709-5

**Published:** 2021-03-17

**Authors:** Fanchang Zeng, Liumei Luo, Daoyuan Li, Juncheng Guo, Min Guo

**Affiliations:** 1 Department of Urology, Hainan General Hospital (Hainan Affiliated Hospital of Hainan Medical University), Haikou, 570311 People’s Republic of China; 2 Department of Science and Education, Hainan General Hospital (Hainan Affiliated Hospital of Hainan Medical University), No. 19, Xiuhua Road, Xiuying District, Haikou, 570311 People’s Republic of China; 3 Department of Urology, Central South University Xiangya School of Medicine Affiliated Haikou Hospital, Haikou, 570208 People’s Republic of China; 4 Psychological Research Center, Hainan General Hospital (Hainan Affiliated Hospital of Hainan Medical University), Haikou, 570311 People’s Republic of China

**Keywords:** Karyopherin alpha 2, Chromobox 8, PR domain zinc finger protein 1, Bladder cancer, Proliferation, Migration, Invasion

## Abstract

**Background:**

Bladder cancer (BCa) is a common malignancy characterized by high heterogeneity, yet the current treatment modalities are limited. The aim of the present investigation was to unravel the functional role of Karyopherin alpha 2 (KPNA2), a tumor facilitator identified in multiple malignancies, in the progression of BCa.

**Methods:**

BCa tissues and adjacent normal tissues were surgically resected and analyzed from patients with BCa to determine the expression profile of KPNA2 and Chromobox 8 (CBX8) by RT-qPCR, Western blot analysis and immunohistochemistry. The relationship among KPNA2, CBX8 and PR domain zinc finger protein 1 (PRDM1) was explored by co-immunoprecipitation and chromatin-immunoprecipitation. The functions of KPNA2, CBX8 and PRDM1 on BCa cell proliferation, migration and invasion were evaluated. Next, a nude mouse model of BCa was established for validating the roles of KPNA2, CBX8 and PRDM1 in vivo.

**Results:**

KPNA2 and CBX8 were highly expressed in BCa and are in association with dismal oncologic outcomes of patients with BCa. KPNA2 promoted nuclear import of CBX8. CBX8 downregulated PRDM1 by recruiting BCOR in the promoter region of PRDM1. Overexpression of KPNA2 promoted the malignant behaviors of BCa cells, which was counteracted by silencing of CBX8. Overexpressing PRDM1 attenuated the progression of BCa by inhibiting c-FOS expression. The tumor-promoting effects of KPNA2 via the PRDM1/c-FOS pathway were also validated in vivo.

**Conclusion:**

Collectively, our findings attached great importance to the interplay between KPNA2 and CBX8 in BCa in mediating the development and progression of BCa, thus offering a promising candidate target for better BCa patient management.

## Background

Bladder cancer (BCa) ranks the 10th most frequently occurring malignancy across the world with approximately 549,000 new cases diagnosed and 200,000 deaths in 2018 [1]. Currently available treatment modalities against BCa include complete resection, chemotherapy, and radiotherapy depending on the different disease subtypes. The identification of biomarker is of great important for different cancers, including colorectal cancer [2], gastric cancer [3], breast cancer [4] and chronic myeloid leukemia [5, 6]. A successful outcome of the treatment for BCa largely depends on early diagnosis and the availability of personalized therapy [7]. Understanding better the molecular features underlying the pathogenesis of BCa could thus offer new opportunities for early diagnosis and novel individualized and targeted therapies [8].

Nuclear-cytoplasmic transport has been indicated to play an important role in mediating cellular functions while the defects in this transport process are implicated in multiple types of cancers [9]. Karyopherin (KPN) nuclear transport receptors have emerged as critical regulators in altering the progression and outcome of cancer [10]. As one of the KPN family members, KPN alpha 2 (KPNA2) has been highlighted to be a biomarker of cancer, which participates in the translocation of diverse proteins associated with carcinogenesis [11]. For instance, KPNA2 has been demonstrated to harbor the oncogenic property of promoting malignant phenotypes of breast cancer cells [12]. The pro-tumor effects of KPNA2 have also been validated in hepatocellular carcinoma (HCC) where KPNA2 mediates the nuclear import of the transcriptional factor pleomorphic adenoma gene 1 (PLAG1) protein [13]. Moreover, there is evidence elucidating the function of aberrantly expressed KPNA2 in BCa by regulating the nuclear transportation of octamer-binding transcription factor 4 [14].

Besides, KPNA2 has been found to interact with Chromobox 8 (CBX8) in human lung adenocarcinoma cancer cells [15]. Expression of CBX8, which is a member of the polycomb family of epigenetic transcription factors, correlates with tumorigenesis in HCC and esophageal squamous cell carcinoma [16–18]. Additionally, the high expression of CBX8 has been elaborated to foreshadow unsatisfactory overall survival and disease-free survival (DFS) of patients with muscle invasive BCa [19]. However, the mechanistic evidence for interplay between KPNA2 and CBX8 in BCa poorly documented. To rectify this, we investigated in the current study how KPNA2 can affect the development and progression of BCa through its regulatory action on the translocation of CBX8. Thus, we endeavored to explore the functional interaction of KPNA2 with CBX8 in BCa in an attempt to unravel the underlying molecular mechanism of this oncogenic pathway.

## Materials and methods

### Ethics statement

The study was conducted with approval of Ethics Committee of Ethics Committee of Hainan General Hospital. Each participant signed written informed consents prior to the study. The animal experiments were approved by the Animal Committee of Hainan General Hospital and conducted in accordance with the relevant regulations and requirements of the Institutional Animal Care and Use Committee (IACUC).

### In silico analysis

The differential analysis of the microarray dataset GSE3167 (n = 50, including 9 normal samples and 41 BCa samples) and GSE7476 (n = 13, including 3 normal samples and 10 BCa samples) obtained from the Gene Expression Omnibus (GEO) database (https://www.ncbi.nlm.nih.gov/gds) was performed using the “limma” package of R language (http://www.bioconductor.org/packages/release/bioc/html/limma.html). The BCa-related data from The Cancer Genome Atlas (TCGA) database (https://portal.gdc.cancer.gov/) were analyzed by means of Gene Expression Profiling Interactive Analysis (GEPIA) (http://gepia.cancer-pku.cn/). The differentially expressed genes (DEGs) were selected with |logFoldChange| > 1.8 and *p* < 0.01 as the threshold. A Venn diagram was plotted to screen out the most significant DEG through intersection of DEGs from the three databases, followed by survival analysis using GEPIA. Then, a protein–protein interaction (PPI) network was established using DEGs that were significantly correlated with patient survival condition with the help of String (https://string-db.org/). The core degree of DEGs in PPI network was analyzed by Cytoscape (https://cytoscape.org/). The downstream regulatory pathway with highest core degree was predicted and verified by GEPIA database and hTFtarget (http://bioinfo.life.hust.edu.cn/hTFtarget#!/).

### Clinical sample collection

In total, 45 patients diagnosed with BCa in Hainan General Hospital from January 2013 to June 2015 were enrolled. The BCa tissues and adjacent non-cancerous tissues (≥ 3 cm from the edge of BCa tissues) were surgically resected, rapidly frozen in liquid nitrogen and stored at − 80 °C before use. BCa tissues were identified by histology and pathology and further classified according to criteria of *The 2004 World Health Organization* grading system. The clinical staging classified based on the International Union Against Cancer (UICC) TNM staging system [20]. Clinical tumor recurrence was recorded when tumor recurrence or metastasis was suggested by clinical and imaging examination. Overall Survival (OS) designated the time period between the date of randomization to the date of death due to any reason. Disease-free survival (DFS) designated the time period between the date of operation to the date of clinical tumor recurrence or death. All clinical data were analyzed using R language software. The data of KPNA2 and CBX8 expression were Log_2_-transformed into a bivariate model. According to the median of the expression of KPNA2 and CBX8, patients with BCa were divided into high expression group and low expression group.

### Lentivirus infection

A human bladder epithelial immortalized cell line SV-HUC-1, Human BCa cell lines (T24 and BIU-87) and HEK293T cells were purchased from American Type Culture Collection (https://www.atcc.org/; Manassas, VA, USA) and cultured in Dulbecco’s Modified Eagle Medium (DMEM, Thermo Fisher Scientific Inc., Waltham, MA, USA) containing 10% fetal bovine serum (FBS) (10100147, Gibco BRL, Invitrogen, CA, USA) at 37 °C with 5% CO_2_.

Cells at the logarithmic growth phase were detached by trypsin and dispersed into single cell suspension (5 × 10^4^ cells/mL), which were then seeded in a 6-well plate (2 mL/well), cultured at 37 °C overnight and infected with different lentivirus. The lentivirus plasmids harboring overexpressed genes (oe-KPNA2, oe-CBX8, oe-PRDM1 and oe-c-FOS) were constructed on LV5-GFP and lentivirus plasmids harboring short hairpin RNA (shRNA) (sh-KPNA2-1, sh-KPNA2-2, sh-CBX8-1, sh-CBX8-2, sh-PRDM1-1 and sh-PRDM1-2) were constructed on pSIH1-H1-copGFP. The lentivirus plasmids harboring shRNA were synthesized by Gene Pharma Co., Ltd., (Shanghai, China). The supernatant was harvested, followed by centrifugation to remove virus particles and enable determination of virus titer determination. At 48 h after infection, the cells were collected and the expression levels of different genes were measured by reverse transcription quantitative polymerase chain reaction (RT-qPCR).

### RT-qPCR

Total RNA was extracted from cells and tissues using Trizol (15596026, Invitrogen) and reversely transcribed to cDNA using a RT kit (RR047A, Takara, Kusatsu, Shiga, Japan). qPCR was performed with SYBR Premix EX Taq kit (RR420A, Takara) in a real-time fluorescence qPCR instrument (ABI7500, ABI, Foster City, CA, USA). The relative expression level was analyzed by 2 ^−ΔΔCt^ method with glyceraldehyde-3-phosphate dehydrogenase (GAPDH) serving as the loading control. Primers for KPNA2, CBX8, PRDM1 and c-FOS were synthesized by Shanghai Sangon Biotech Co., Ltd., (Shanghai, China) as shown in Table 1.

**Table 1:** Primer sequences for RT-qPCR

Gene	Primer sequences
KPNA2 (human)	F: 5′-CTGGGACATCAGAACAAACCAAG-3′
	R: 5′-ACACTGAGCCATCACCTGCAAT-3′
CBX8 (human)	F: 5′-CCTTCGAAACATGGGTTTGT-3′
	R: 5′-CTGGGCTTGTCATCCACTCT-3′
PRDM1 (human)	F: 5′-CCCTCATCGGTGAAGTCTA-3′
	R: 5′-ACGTAGCGCATCCAGTTG-3′
c-FOS (human)	F: 5′-AGAATCCGAAGGGAAAGGAA-3′
	R: 5′-CTTCTCCTTCAGCAGGTTGG-3′
U6	F: 5′-CGGCGGTCGTGAAGCGTTCCAT-3′
	R: 5′-CCAGTGCAGGGTCCGAGGTAT-3′
GAPDH	F: 5′-CACCCACTCCTCCACCTTTG-3′
	R: 5′-CCACCACCCTGTTGCTGTAG-3′

RT-qPCR: reverse transcription quantitative polymerase chain reaction; F: forward; R: reverse; KPNA2: karyopherin α2; CBX8: Chromobox 8; PRDM1: PR domain zinc finger protein 1; c-FOS: Cellular oncogene fos; GAPDH: glyceraldehyde-3-phosphate dehydrogenase

### Western blot analysis

Total protein was extracted from cells and tissues by high-effective Radio Immunoprecipitation Assay Lysis Buffer (R0010, Solarbio Life Sciences Co., Ltd., Beijing, China) in strict accordance with the manual. Nucleoprotein and cytoplasmic protein were prepared using cytoplasm and nucleus protein extraction kits (Thermo Fisher Scientific). The protein concentration was determined using a bicinchonininc acid protein assay kit (20201ES76, Yeasen Biotech Co., Ltd., Shanghai, China). The proteins were separated by 10% sodium dodecylsulphate-polyacrylamide gel electrophoresis and wet-transferred onto the polyvinylidene fluoride membrane (Millipore, Billerica, MA, USA) in a wet manner. The membrane was blocked in 5% bovine serum albumin at room temperature for 1 h, and then probed with the following diluted primary antibodies: rabbit polyclonal antibodies to KPNA2 (ab84440, 1:1000, Abcam Inc., Cambridge, UK), CBX8 (ab70796, 1:500, Abcam), PRDM1 (ab119401, 1:1000, Abcam), c-FOS (ab190289, 1:2000, Abcam), and GAPDH (ab181602, 1:2000) at 4 °C overnight, and then re-probed with horseradish peroxidase-conjugated goat anti-rabbit immunoglobulin G (ab205718, 1:10000, Abcam) at room temperature for 1 h. The protein bands were visualized by VILBER FUSION FX5 (Vilber Lourmat, Paris, France) and analyzed by ImageJ 1.48u software (National Institutes of Health, Bethesda, Maryland, USA). The relative expression was calculated as the ratio of gray value of target band to that of the internal reference (GAPDH).

### Immunohistochemistry (IHC)

BCa tissue blocks were fixed in 4% paraformaldehyde-phosphate buffer for 12 h and cut into slices of 4 μm-thickness. The tissue slides were dewaxed with xylene, hydrated by gradient ethanol, boiled in 0.01 M citric acid buffer solution for 15–20 min and cooled down to room temperature. The tissue slides were incubated with 50 μL primary rabbit antibodies to KPNA2 (ab84440, 1:200, Abcam) and CBX8 (ab182627, 1:50, Abcam) at room temperature for 1 h and with secondary antibody goat anti-rabbit immunoglobulin G (IgG; ab6728, 1:1000, Abcam) at room temperature for another 1 h. Then the slides were incubated with streptavidin peroxidase at 37 °C for 30 min. Color was developed using 3,3-*N*-diaminobenzidine (DAB) for 5 min and reaction was terminated by washing under running water for 10 min. Hematoxylin was applied for 2-min counterstaining, followed by hydrochloric acid–ethanol for differentiation. The sample was dehydrated, cleared, sealed and observed under an inverted microscope.

### Co-immunoprecipitation (Co-IP)

T24 cells were collected and lysed. The polyclonal antibodies to KPNA2 (ab70160, 1:100, Abcam) and CBX8 (ab182627, 1:100, Abcam) were used for Co-IP with reference to the instructions of the Pierce Classic IP Kit (Thermo Fisher Scientific). In brief, protein extracts were incubated with specific antibody or IgG (negative control) at 4 °C overnight. The immune complex was collected after incubated with protein A agarose for 2 h at 4 °C. Following 3 washes using protein lysis buffer (0.7 mL), the precipitate was boiled, cooled, and analyzed by 10–12% SDS-PAGE, followed by Western blot analysis.

### Chromatin immunoprecipitation (ChIP)

BCa cells were fixed with formaldehyde for 10 min to generate DNA–protein crosslink and then disrupted by Ultrasonic Cell Disruptor (UP-250, Scientz, Ningbo, Zhejiang, China), 10 s each at an interval of 10 s, 15 cycles in total. The supernatant was collected through centrifugation at 4 °C for 10 min at 12,000 rpm and sub-packed into 2 tubes. One tube was added with rabbit antibody to IgG (ab109489, 1: 300, Abcam) as NC and the other tube was added with antibody to CBX8 (ab182627, 1: 50, Abcam) for incubation at 4 °C overnight. The DNA–protein complex was precipitated by Protein Agarose/Sepharose. The supernatant was removed through centrifugation at 12,000×*g* for 5 min. The non-specific complex was washed, followed by de-crosslinking at 65 °C overnight. DNA fragments were purified and retrieved by phenol/chloroform. The binding between CBX8 and PRDM1 was detected by RT-qPCR using PRDM1 specific primers.

### Immunofluorescence

The cells were fixed in 4% paraformaldehyde for 15 min, penetrated by 0.5% Triton X-100 (Sangon) at room temperature for 20 min, and blocked in 5% normal goat serum (Solarbio Science & Technology Co., Ltd., Beijing, China) at room temperature for 30 min. Then the cells were incubated with rabbit anti-KPNA2 (ab70160, 1:100, Abcam) and CBX8 (ab182627, 1:100, Abcam) in a humidified box at 4 °C overnight. Then the cells were incubated with Alexa Fluor 647-labeled donkey anti-rabbit IgG (ab150075, 1:400, Abcam) for 1 h at 37 °C in the dark. The nucleus was stained by 4’,6-diamidino-2-phenylindole (BioDee Biotechnology Co., Ltd., Beijing, China) in the dark for 5 min. The cells were finally sealed by fluorescent mounting media and images were acquired under a fluorescence microscope (IX73, Olympus, Tokyo, Japan).

### Cell counting kit-8 (CCK-8) assay

The cells were seeded in a 96-well plate at a density of 2 × 10^3^ cells/well. After 24 h-infection, cell proliferation was evaluated with a CCK-8 kit (Dojindo Laboratories, Kumamoto, Japan). The optical density value at 450 nm was measured by a microplate reader (Bio-Rad, Hercules, CA, USA).

### 5-ethynyl-2′-deoxyuridine (EdU) assay

The cells at the logarithmic growth phase were seeded in a 96-well plate at a density of 2 × 10^3^ − 4 × 10^4^ cells/well and cultured overnight. 48 h after infection, cells were labeled with EdU by incubated with EdU (100 μL/well) for 2-h. Cells were fixed with cell fixative (100 μL/well) for 30-min at room temperature and incubated with 2 mg/mL glycine for another 5 min. The cells were further incubated for 10 min with phosphate buffer saline (PBS) containing 0.5% TritonX-100 (100 μL/well), stained with 1 × Apollo for 30 min and with 1 × Hoechst 33342 for 30 min (100 μL/well) at room temperature in the dark. Then, fluorescent mounting media (100 μL/well) was used for sealing. The number of EdU-labeled cells in 3 randomly-selected fields was counted under the fluorescence microscope. EdU labeling rate (%) = the number of positive cells/(the number of positive cells + the number of negative cells) × 100%.

### Terminal deoxynucleotidyl transferase dUTP nick end labeling (TUNEL) staining

BCa tissues were fixed in 4% paraformaldehyde overnight, embedded with paraffin and sliced into 5-mm sections. Five sections were dewaxed, hydrated, and incubated with 50 μL of 1% proteinase K dilution at 37 °C for 30 min. The activity of endogenous peroxidase (POD) was blocked by incubated with methanol solution supplemented with 0.3% H_2_O_2_ at 37 °C for 30 min. TUNEL reaction solution was added and incubated with sections in a humidified box at 37 °C for 1 h in the dark. Then, 50 μL Converter-POD was added and incubated with the sections in a wet box at 37 °C for another 30 min. Color was visualized by incubation with 2% DAB for 15-min at room temperature. The reaction was terminated by adding distilled water. The sections were then counterstained by hematoxylin, followed by dehydration using ethanol of ascending concentrations and xylene clearing. After sealed with neutral gum, sections were observed under a microscope (40×) in 10 randomly-selected fields. The brown nuclei were indicative of apoptotic positive cells and blue nuclei were indicative of normal cells. The apoptosis rate = the number of apoptotic cells/the number of normal cells.

### Transwell assay

Invasion assay was performed. The extracellular matrix (ECM) gel (E1270-1ML, Sigma-Aldrich Chemical Company, St Louis, MO, USA) was diluted to 1 mg/mL with serum-free medium. The polycarbonate membrane in the apical chambers was supplemented with 40 μL ECM gel and incubated for 5 h at 37 °C with 5% CO_2_ for polymerization, which was then incubated with DMEM (70 µL/chamber) for 0.5 h at 37 °C for rehydration. BCa cells were starved for 24 h in serum free DMEM and re-suspended in serum free DMEM to make the final cell concentration 2.5 × 10^5^ cells/mL. Then, 0.2 mL cell suspension was added into apical Transwell chambers with hydrated basement membrane while the basolateral chambers were added with 700 µL pre-cold DMEM containing 10% FBS, followed by incubation under saturated humidity at 37 °C with 5% CO_2_ for 24 h. With the removal of chambers, the cells on the chambers and basement membrane were wiped out using wet cotton swab, fixed with methanol for 30 min, stained with 0.1% crystal violet for 20 min and aired dried. An inverted microscope (CKX53, OLYMPUS) was used for observation at 200 × within 5 randomly selected fields (200×). The number of cells that crossed through the membrane was counted.

Migration assay was conducted. Briefly, the cells were transfected with plasmid DNA for 24 h and then digested into a single cell suspension. Cell concentration was adjusted to 1 × 10^6^ cells/mL in medium containing 1% FBS. A 24-well plate was added with 10% FBS-containing complete medium at 600 μL/well. The Transwell chamber was placed on the plate and added with the adjusted amount of cell suspension at 100 μL per chamber, followed by incubation at 37 °C for 24 h. The chamber was removed and the medium and non-migrated cells in the chamber were wiped off using a cotton swab, then fixed with a fixative with a ratio of methanol and acetic acid of 3: 1 for 15–30 min, and air-dried. The chamber was stained with 1% crystal violet for 20 min and cell migration was observed under an inverted microscope (CKX53, OLYMPUS, Japan), photographed and counted. Each experiment was repeated three times.

### Scratch test

The transfected cells were seeded in a 6-well plate at 5 × 10^5^ cells/well. When the cell growth and confluence reached about 90%, the axis of the hole was gently scratched using a sterile pipette tip. After washing with PBS to remove floating cells, the cells were added with serum-free medium and continued to culture for 0.5–1 h to recover the cells. The cells were photographed at 0 h and 24 h, respectively, and the migration distance of the cells was measured with Image-Pro Plus Analysis software (Media Cybernetics, Silver Spring, MD, USA), and the value was averaged.

### Xenograft tumor in nude mice

In total, 36 BALA/C nude mice (age: 4–5 weeks, weight: 16–18 g) were purchased from Hunan SJA Laboratory Animal Co., Ltd., (Changsha, Hunan, China) (http://www.hnsja.com/), and raised under specific pathogen free environment. Every 12 mice were inoculated with BCa cells (5 × 10^3^ cells/mouse) harboring oe-NC, oe-KPNA2 or oe-KPNA2 + oe-PRDM1. The volume of xenograft tumors was measured on the 10th day after inoculation and calculated using the formula that V (mm^3^) = (A × B^2^)/2, where A is the long diameter and B is the short diameter. The curve displaying the average volume at each time point was plotted.

### Statistical analysis

Data analysis was performed using SPSS 21.0 software (IBM, Armonk, NY, USA). Measurement data were expressed as the mean ± standard deviation unless otherwise indicated. Data in normal distribution and homogeneity of variance were compared by paired *t*-test within the group, by independent sample *t*-test between two groups and by one-way analysis of variance (ANOVA) among multiple groups, followed by Tukey’s post hoc test. Data comparison at different time points was analyzed by repeated measures ANOVA, followed by Tukey’s post hoc test. Kaplan–Meier was introduced to plot a survival curve, while Log-rank test was used for differential analysis. Cox proportional hazard regression model was used to evaluate the individual variables influencing the survival risk of BCa. Wald’s test was used to determine the statistical significance of univariate and multivariate Cox regression models. A *p* value of < 0.05 was considered statistically significant.

## Results

### High expression of KPNA2 and CBX8 in BCa was correlated with poor prognosis

**Fig. 1:**
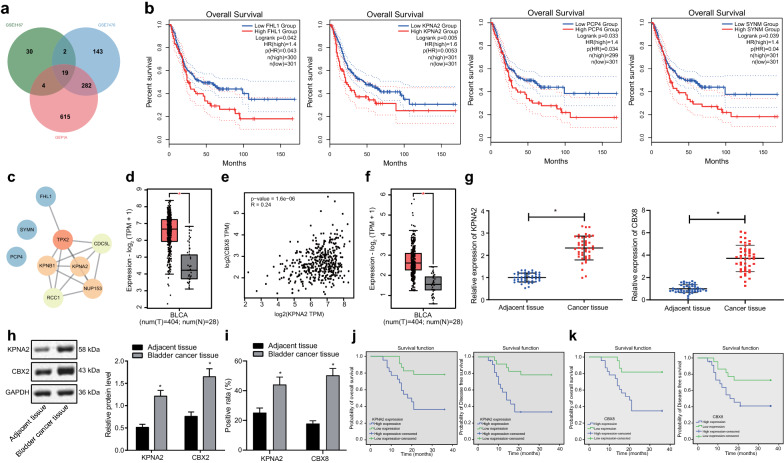
High expression of KPNA2 and CBX8 in BCa is associated with dismal oncological outcomes of patients with BCa. **a** Venn diagram illustrating DEGs in BCa through microarray dataset GSE3167 and GSE7476 and TCGA database by GEPIA. **b** The survival curves displaying the correlation between the expression of KPNA2, PCP4, SYNM or FHL1 and survival of patients with BCa. **c** The PPI networks of KPNA2, PCP4, SYNM and FHL1. The deeper red color suggests higher core degree and the deeper blue color suggests lower core degree. **d** The box plot of KPNA2 expression in BCa samples (left red box) and normal samples (right grey box) according to GEPIA; *p < 0.05 vs. normal samples. **e** The correlation analysis between expression of KPNA2 and CBX8 in BCa according to GEPIA (*p* = 1.6e−06). **f** The box plot of CBX8 expression in BCa samples (left red box) and normal samples (right grey box) according to GEPIA; *p < 0.05 vs. normal samples. **g** The expression of KPNA2 and CBX8 in BCa tissues and adjacent non-cancerous tissues determined by RT-qPCR; *p < 0.05 vs. adjacent non-cancerous tissues. **h** The expression of KPNA2 and CBX8 in BCa tissues and adjacent non-cancerous tissues determined by Western blot analysis; *p < 0.05 vs. adjacent non-cancerous tissues. **i** The expression of KPNA2 and CBX8 in BCa tissues and adjacent non-cancerous tissues identified by IHC (× 100); *p < 0.05 vs. adjacent non-cancerous tissues. **j** The correlation between KPNA2 expression and survival condition analyzed by Kaplan–Meier. **k** The correlation between CBX8 expression and survival condition analyzed by Kaplan–Meier. n = 45. Data comparison between BCa tissues and adjacent non-cancerous tissues was analyzed by paired *t*-test

Through differential analysis on GEO microarray dataset GSE3167 and GSE7476 combined with GEPIA on TCGA database, 55,446 and 920 DEGs were obtained respectively and 19 DEGs were further revealed in the intersection by means of Venn diagram (Fig. 1a). Subsequently, a survival curve displaying the correlation between the expression profile of 19 DEGs and survival condition of patients with BCa showed 4 significantly correlated DEGs, KPNA2, PCP4, SYNM and FHL1, among which KPNA2 had the most significant correlation (Fig. 1b). Following establishment of a PPI network for the above 4 genes using String, Cytoscape was introduced for image processing, which revealed KPNA2 to be the gene with highest core degree (Fig. 1c, Table 2). Besides, KPNA2 was revealed as a significantly upregulated gene in BCa according to the GEPIA results (Fig. 1d, Table 3).

**Table 2:** Survival analysis of 19 DEGs

Gene	*p* (HR)	HR	Gene	*p* (HR)	HR
KPNA2	0.0053	1.6	ACTG2	0.099	1.3
PCP4	0.034	1.4	ACTC1	0.1	1.3
SYNM	0.04	1.4	MYH11	0.11	1.3
FHL1	0.043	1.4	CFD	0.21	1.2
SRPX	0.051	1.4	MYL9	0.22	1.2
TNS1	0.056	1.4	TOP2A	0.35	0.85
SPARCL1	0.073	1.3	LMOD1	0.54	1.1
TAGLN	0.083	1.3	CKS2	0.59	1.1
CNN1	0.088	1.3	MYLK	0.62	1.1
DES	0.089	1.3			

HR: Hazard Ratio (HR > 1 indicates high risk gene, HR < 1 indicates low risk gene); DEG: differentially expressed genes; KPNA2: karyopherin α2; PCP4: Purkinje cell protein 4; SYNM: Synemin; FHL1: Four and a half LIM domain protein 1; SRPX: sushi repeat-containing protein X; TNS1: tensin-1; SPARCL1: Secreted protein acidic and rich in cysteine like-1; TAGLN: transgelin; CNN1: calponin-1; DES: desulfhydrase

**Table 3:** The core degree of significant risk genes

Gene	Degree
FHL1	1
PCP4	0
SYNM	0
KPNA2	5

FHL1: Four and a half LIM domain protein 1; PCP4: Purkinje cell protein 4; SYNM: Synemin; KPNA2: karyopherin α2

Further GEPIA results demonstrated a positive correlation between the expression of KPNA2 and CBX8 in BCa samples (Fig. 1e). CBX8 was confirmed to be another upregulated gene in BCa (Fig. 1f), which is highly suggestive of the promotive effects induced by the interplay between KPNA2 and CBX8 on the progression of BCa.

Subsequently, RT-qPCR (Fig. 1g), Western blot analysis (Fig. 1h) and IHC (Fig. 1i) were performed to determine the expression profile of KPNA2 and CBX8 in BCa tissues collected from patients with BCa. We saw significantly higher expression of KPNA2 and CBX8 in BCa tissues than in adjacent non-cancerous tissues was observed. The correlation analysis between KPNA2 and CBX8 expression and prognosis of BCa patients through follow-up visits showed an inverse relationship, whereby patients with highly expressed KPNA2 and CBX8 exhibited poor overall survival and DFS (Fig. 1j, k). The expression of KPNA2 and CBX8 was correlated with the pathological grade, clinical stage, metastasis, and recurrence of the tumor (*p *< 0.05), but not correlated with age, gender, smoking history, and gross hematuria (*p *> 0.05) (Table 4). Taken together, these findings indicated that KPNA2 and CBX8 were highly expressed in BCa tissues and served as adverse prognostic biomarkers.

**Table 4:** Correlation between the expression of KPNA2 and CBX8 in tissues and the characteristics of clinical cases

Group	Cases	KPNA2 expression		*p*	CBX8 expression		*p*
		Downregulate	Upregulate		Downregulate	Upregulate	
Age							
> 60	15	5	10	> 0.05	7	8	> 0.05
≤ 60	30	17	13		16	14	
Gender							
Male	35	19	16	> 0.05	18	17	> 0.05
Female	10	3	7		5	5	
TNM staging							
G1	17	5	12	< 0.05	5	12	< 0.05
G2–G3	28	17	11		18	10	
Clinical staging							
T1	24	4	20	< 0.05	8	16	< 0.05
T2–T3	21	18	3		15	6	
Smoking history							
Yes	28	15	13	> 0.05	13	15	> 0.05
No	17	7	10		10	7	
Gross hematuria							
Yes	30	15	15	> 0.05	16	14	> 0.05
No	15	7	8		7	8	
Metastasis							
Yes	14	11	3	< 0.05	11	3	< 0.05
No	31	11	20		12	19	
Recurrence							
Yes	19	15	4	< 0.05	14	5	< 0.05
No	26	7	19		9	17	

### KPNA2 promoted nuclear import of CBX8

**Fig. 2:**
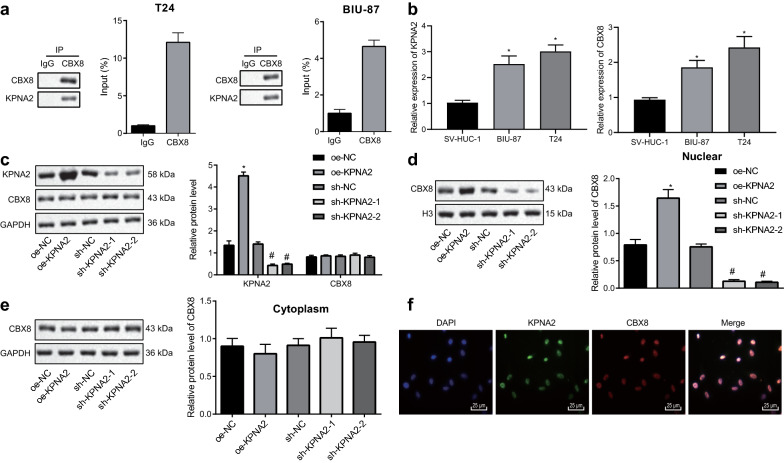
KPNA2 assists nucleus shuttling of CBX8. **a** The binding between KPNA2 and CBX8 in T24 and BIU-87 cells detected by Co-IP assay. **b** The PNA2 and CBX8 mRNA expression detected in T24 and BIU-87 cell lines using RT-qPCR. **c** The expression of KPNA2 and CBX8 in BCa cells in response to overexpressing/silencing KPNA2 determined by Western blot analysis. **d** The expression of CBX8 in the nucleus of BCa cells in response to overexpressing/silencing KPNA2 determined by Western blot analysis. **e** The expression of CBX8 in the cytoplasm of BCa cells in response to overexpressing/silencing KPNA2 determined by Western blot analysis. **f** The nucleus accumulation of KPNA2 and CBX8 in T24 cells by immunofluorescence staining. *p < 0.05 vs. T24 cells treated with oe-NC; ^#^*p* < 0.05 vs. T24 cells treated with sh-NC

To investigate whether the nuclear translocation of CBX8 in BCa was mediated by KPNA2, KPNA2 antibody was introduced for Co-IP assay. CBX8 was detected in KPNA2 antibody-enriched precipitate of the BCa cell lines, BIU-87 and T24 (Fig. 2a). Moreover, KPNA2 antibody could enrich more CBX8 in T24 cells. Then, KPNA2 and CBX8 mRNA expression was detected in T24 and BIU-87 cell lines using RT-qPCR (Fig. 2b). The results displayed that KPNA2 and CBX8 mRNA expression was increased in T24 and BIU-87 cells relative to SV-HUC-1 cells, and their levels were most significantly upregulated in T24 cells. Therefore, T24 cells were selected for subsequent use. Next, in vitro models were developed when KPNA2 was overexpressed or knocked down in T24 cells. According to Western blot analysis, KPNA2 expression was upregulated/downregulated in response to overexpression/knockdown of KPNA2 (Fig. 2c). Following isolation of nucleoproteins and cytoplasmic proteins, Western blot analysis showed that overexpressing KPNA2 could significantly increase the expression of KPNA2 and CBX8 in the nucleus but slightly decrease the expression of CBX8 in the cytoplasm while opposite results were observed after silencing KPNA2 (Fig. 2d, e). Observation through immunofluorescence microscopy revealed accumulation of KPNA2 and CBX8 in the nucleus after overexpression of KPNA2 (Fig. 2f). To conclude, it was suggested that KPNA2 acted as positive regulator for the nuclear import of CBX8 through its binding with CBX8.

### KPNA2 mediated the proliferation, migration, and invasion of BCa cells through regulating CBX8

**Fig. 3:**
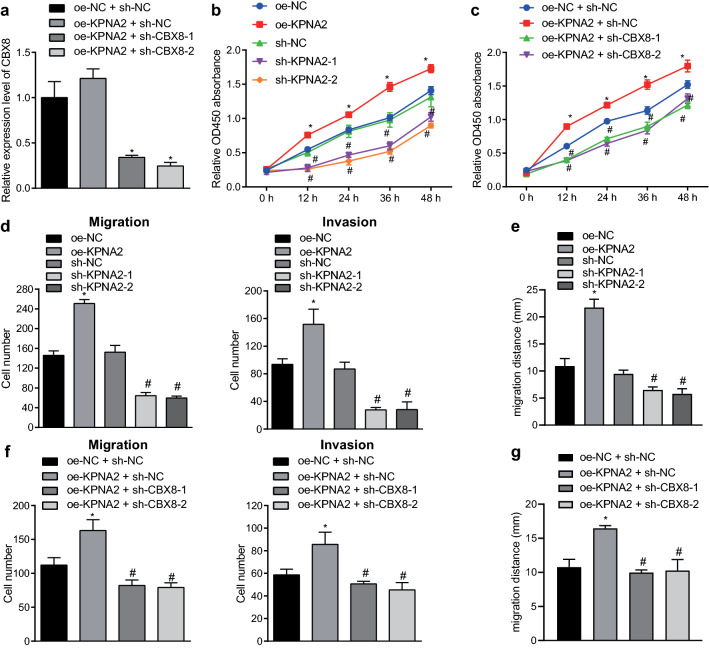
CBX8 is required for the mediatory function of KPNA2 in BCa cell proliferation, migration and invasion. **a** The expression of CBX8 determined by RT-qPCR. *p < 0.05 vs. T24 cells treated with oe-KPNA2 + sh-NC. **b**, **c** The proliferation of BCa cells detected by CCK-8 assay. **d** The number of migratory and invasive BCa cells detected by Transwell assay (×200). **e** Migration of BCa cells detected by scratch test (×200). **f** The number of migratory and invasive BCa cells detected by Transwell assay (×200). **g** Migration of BCa cells detected by scratch test (×200). *p < 0.05 vs. T24 cells treated with oe-NC (panels **b** and **d**) or oe-NC + sh-NC (panels **c** and **e**). ^#^*p* < 0.05 vs. T24 cells treated with sh-NC (panels **b** and **d**) or oe-KPNA2 + sh-NC (panels **c** and **e**)

The investigation focus was then shifted to an exploration of the functional significance of interaction between KPNA2 and CBX8 in BCa. Firstly, KPNA2 was overexpressed and CBX8 was silenced in T24 cells. RT-qPCR showed that sh-CBX8 obviously reduced the expression of CBX8 in the presence of oe-KPNA2 (Fig. 3a). Then, the proliferative capability of tumor cells was measured by CCK-8 assays (Fig. 3b, c), migration and invasion were evaluated by Transwell assay and scratch test (Fig. 3d–g). The proliferation, migration, and invasion of BCa cells were significantly retarded after inhibition of KPNA2 but accelerated after overexpression of KPNA2 (Fig. 3b, d, e). Besides, silencing of CBX8 could significantly counteracted the effects of KPNA2 overexpression on the proliferation, migration, and invasion of BCa cells (Fig. 3c, f, g). In summary, the results elucidated that the mediation of KPNA2 in BCa cell proliferation, migration and invasion was dependent on CBX8.

### CBX8 downregulated PRDM1 by recruiting BCOR in the promoter region of PRDM1

**Fig. 4:**
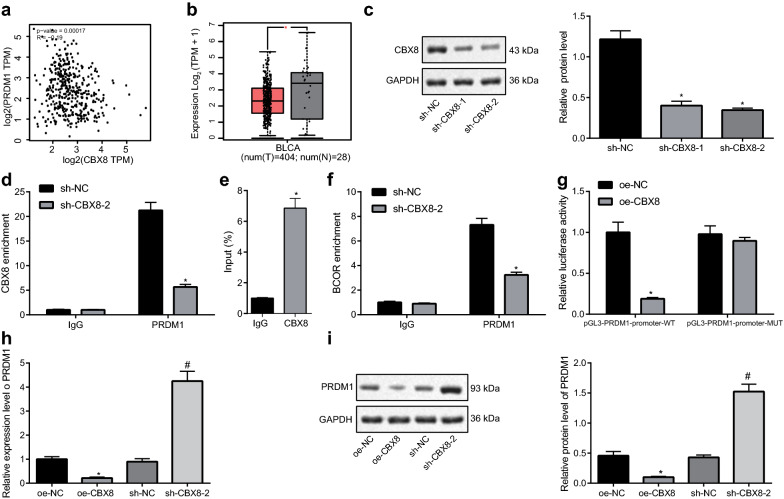
CBX8 recruits PRC1-BCOR to the PRDM1 promoter region to downregulate PRDM1 expression. **a** The correlation analysis between expression of CBX8 and PRDM1 in BCa according to GEPIA (*p* = 0.00017). **b** The box plot of PRDM1 expression in BCa samples (left red box) and normal samples (right grey box) according to GEPIA. *p < 0.05 vs. normal samples analyzed by paired *t*-test. **c** The silencing efficiency of sh-CBX8 determined by Western blot analysis. **d** The enrichment of CBX8 in the PRDM1 promoter region detected by ChIP assay. **e** The interaction between CBX8 and BCOR detected by co-IP. **f** The enrichment of BCOR in the PRDM1 promoter region detected by ChIP. **g** The relative luciferase activity determined by dual luciferase reporter assay. **h** The expression of PRDM1 in response to CBX overexpression/knockdown determined by RT-qPCR. **i** The expression of PRDM1 in response to CBX overexpression/knockdown determined by Western blot analysis. *p < 0.05 vs. T24 cells treated with sh-NC (panels **c**, **d**, **f** and **g**) or T24 cells treated with oe-NC (panels **h** and **i**); ^#^*p* < 0.05 vs. T24 cells treated with sh-NC (panels **h** and **i**)

A previous report had elucidated that CBX8 can bind to polycomb repressive complex-BCL6 corepressor (BCOR) to suppress the transcription of positive regulatory domain 1 (PRDM1), by which lymphomagenesis is facilitated [21]. In this part of the study, we intended to explore whether PRDM1 functioned downstream of CBX8 in BCa. An inverse relationship between the expression of CBX8 and PRDM1 in BCa was revealed from GEPIA (Fig. 4a) and PRDM1 was found to be poorly expressed in BCa (Fig. 4b). To determine the regulatory mechanism of CBX8 expression in BCa cells, CBX8 was silenced in T24 cells. Western blot analysis confirmed that CBX8 expression was remarkably decreased by both sh-CBX8-1 and sh-CBX8-2, and sh-CBX8-1 was selected for subsequent experiments because of its higher silencing efficiency (Fig. 4c). The enrichment of CBX8 in the PRDM1 promoter region was then detected by ChIP assay, which revealed notably reduced enrichment of CBX8 in the promoter region of PRDM1 in response to CBX8 knockdown (Fig. 4d). For further investigation on whether CBX8 could bind to BCOR in BCa cells, a Co-IP assay was conducted in T24 cells, which showed that BCOR was enriched with CBX8 antibody compared with IgG (Fig. 4e). Then, the binding of BCOR in the PRDM1 promoter region was detected by ChIP, which showed that BCOR enrichment was dramatically reduced in the presence of sh-CBX8-1, thus suggesting that CBX8 was necessary for BCOR recruitment in the PRDM1 promoter (Fig. 4f). Further dual luciferase reporter assay demonstrated that silencing CBX8 could enhance the luciferase activity of PRDM1-promoter-WT (Fig. 4g). As shown in Fig. 4h, i, PRDM1 expression sharply decreased in response to CBX8 overexpression, and increased in response to CBX8 knockdown as measured by RT-qPCR and Western blot analysis. Collectively, CBX8 was validated to inhibit PRDM1 expression through recruitment of BCOR to the PRDM1 promoter region.

### PRDM1 contributed to the progression of BCa by inhibiting c-FOS expression

**Fig. 5:**
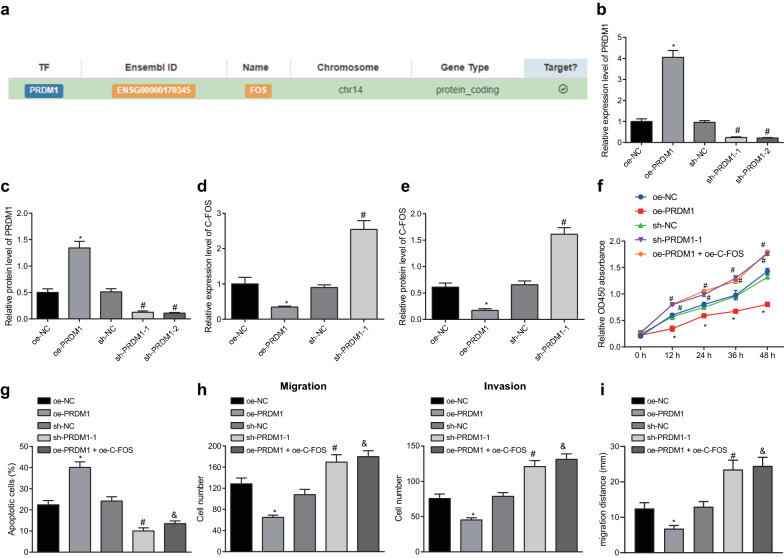
The interaction between PRDM1 and c-FOS facilitates the malignant behaviors of BCa cells. **a** The targeting relationship between PRDM1 and c-FOS verified by hTFtarget. **b** The expression of PRDM1 determined by RT-qPCR. **c** The expression of PRDM1 determined by Western blot analysis. **d** The expression of c-FOS determined by RT-qPCR. **e** The expression of c-FOS determined by Western blot analysis. **f** The proliferation of BCa cells detected by CCK-8 assay. **g** The apoptosis of BCa cells detected by TUNEL staining (×200). **h** The number of migratory and invasive BCa cells detected by Transwell assay (×200). **i** migration of BCa cells detected by scratch test (×200). *p < 0.05 vs. T24 cells treated with oe-NC. ^#^*p* < 0.05 vs. T24 cells treated with sh-NC. ^&^*p* < 0.05 vs. T24 cells treated with oe-PRDM1

Since PRDM1 was poorly expressed in BCa, we overexpressed PRDM1 in T24 cells. Results showed that upregulating PRDM1 suppressed cell proliferation, induced cell apoptosis and inhibited cell migration and invasion. As revealed from the hTFtarget database, PRDM1 could mediate the expression of c-FOS as a transcription factor (Fig. 5a). We overexpressed or silenced PRDM1 expression in T24 cells and then the transfection efficiency of PRDM1 were successfully validated by RT-qPCR (Fig. 5b) and Western blot analysis (Fig. 5c), among which the sh-PRDM1-1 with the most silencing effect was selected for subsequent use. Next, we overexpressed PRDM1 in T24 cells, and performed RT-qPCR (Fig. 5d) and Western blot analysis (Fig. 5e), which showed that c-FOS expression was significantly diminished by oe-PRDM1. Furthermore, CCK-8 assay (Fig. 5f), TUNEL staining (Fig. 5g), Transwell assay (Fig. 5h) and scratch test (Fig. 5i) revealed that overexpressing c-FOS reversed the effects of upregulation of PRDM1 on cell proliferation, apoptosis, migration, and invasion. To conclude, PRDM1 downregulated c-FOS expression in contribution to the progression of BCa. Collectively, silencing of PRDM1 promotes the expression of C-FOS to promote the development of BCa.

### KPNA2/PRDM1/c-FOS axis promoted tumor growth of BCa in vivo

**Fig. 6:**
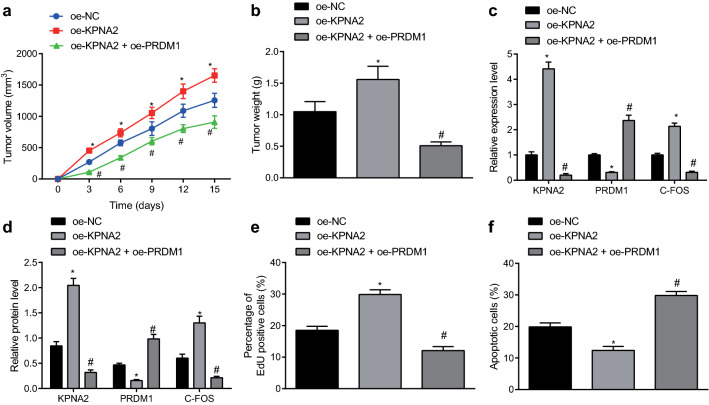
KPNA2 contributes to the xenograft tumor growth of BCa in vivo by mediating the PRDM1/c-FOS pathway. **a** The curve displaying tumor volume at various time points. **b** The representative images of resected tumors and curve displaying tumor weight at various time points. **c** The expression of KPNA2, PRDM1 and c-FOS in transplanted tumor tissues determined by RT-qPCR. **d** The expression of KPNA2, PRDM1 and c-FOS in transplanted tumor tissues determined by Western blot analysis. **e** The tumor cell proliferation detected by EdU assay (×400). **f** The tumor cell apoptosis detected by TUNEL assay (×200). n = 12. * *p* < 0.05 vs. nude mice bearing T24 cells treated with oe-NC. ^#^*p* < 0.05 vs. nude mice bearing T24 cells treated with oe-KPNA2

Finally, we endeavored to reproduce the effects of the KPNA2/PRDM1/c-FOS axis in vivo through subcutaneous implantation of T24 cells with overexpressed KPNA2/PRDM1 into BALA/C nude mice. Results revealed that tumor volume (Fig. 6a) and weight (Fig. 6b) were significantly enhanced in the presence of oe-KPNA2, whereas KPNA2 and c-FOS expression was upregulated and PRDM1 expression was downregulated (Fig. 6c, d) accompanied by accelerated cell proliferation (Fig. 6e) and suppressed cell apoptosis (Fig. 6f). Furthermore, PRDM1 overexpression could counteract the pro-tumor effects of KPNA2. To sum up, KPNA2 was proved to promote tumorigenesis of BCa in vivo through the PRDM1/c-FOS pathway.

## Discussion

In recent years, research attention has been increasingly paid to the diagnostic, prognostic, and therapeutic significance of nuclear transportation in malignancies. In this context, the KPN superfamily stands out as a group of soluble transport receptors [22]. During the current investigation, we selected KPNA2 as the research target to assess its prognostic value in BCa. Collectively, the experimental data demonstrated a detrimental contribution of KPNA2 to BCa in enhancing the proliferation, migration, and invasion of BCa cells in both in vitro and in vivo. This detrimental effect was obtained by assisting the nuclear shuttling of CBX8 and therefore regulating the PRDM1/c-FOS pathway. Results of our study suggested that high expression of CBX8 played a critical oncogenic role in the aggressiveness of urothelial carcinoma cells of the bladder by promoting cancer cell proliferation. These results suggested that CBX8 could be used as a novel biomarker predictive for muscle invasive BCa in patients.

An initial finding of our study was that KPNA2 and CBX8 were found to be highly expressed in BCa tissues and cell, both of which serve as independent risk factor for overall survival and DFS of patients with BCa, indicating the clinical significance of these proteins. Largely in agreement with our present results, high expression of KPNA2 has been identified in patients with BCa as an independent biomarker for dismal outcomes in BCa patients, and to be highly suggestive of greater risk for visceral metastasis and bladder recurrence after treatment [23, 24]. In the context of colorectal cancer, non-small-cell lung cancer and HCC, patients with high expression of KPNA2 have been reported to usually have generally unsatisfactory overall survival condition [25–27]. Besides, patients with muscle invasive BCa exhibited high expression of CBX8 in association with reduced overall survival and DFS [19]. Accumulating evidence has revealed the involvement of CBX8 in regulatory work of different malignancies, including breast cancer, esophageal carcinoma, and HCC [28–30]. Moreover, upregulated CBX8 expression exerts oncogenic function on the aggressiveness of BCa cells by promoting the malignant prototype by repressing the p53 pathway [19] supporting the generalization of our findings. On the other hand, inhibition of CBX8 in T24 cells could counterbalance the pro-tumor effects of KPNA2, suggesting that the oncogenic role of KPNA2 in BCa was dependent on CBX8.

Furthermore, silencing of KPNA2 led to suppressed cell proliferation, migration, and invasion in vitro as well as inhibited carcinogenic potency in vivo. Concordant with our study, KPNA2 is known for its capacity to shuttle cargo proteins related to tumorigenicity between nucleus and cytoplasm [31, 32]. Concordantly, a previous study has confirmed the crosstalk between KPNA2 and OCT in BCa, showing that nuclear transportation of OCT can be inhibited by silencing KPNA2 [14]. Similar to our findings, the nuclear import of PLAG1 has been illustrated to be aided by KPNA2 in hepatocellular carcinoma (HCC) and PLAG1 seemed to be partly responsible for the functional role of KPNA2 in HCC [13]. Also, the significance of KPNA2 as a nucleocytoplasmic transport protein in breast cancer has been recognized by virtue of its mediation of the subcellular localization of proteins associated with DNA damage response in breast cancer [33].

Additionally, our study showed CBX8 downregulated the PRDM1 expression by recruiting BCOR to the PRDM1 promoter region. Likewise, the interplay between CBX8 and BCOR has been documented to play a role in lymphomagenesis [21]. Downregulation of PRDM1 has been elucidated to correspond to poor prognosis and increased malignant phenotypes in lung cancer and colon cancer [34, 35]. Moreover, silencing of PRDM1 exerted promoting effects on the development of BCa by upregulating c-FOS, which is significantly upregulated in BCa [36–38].

## Conclusion

**Fig. 7:**
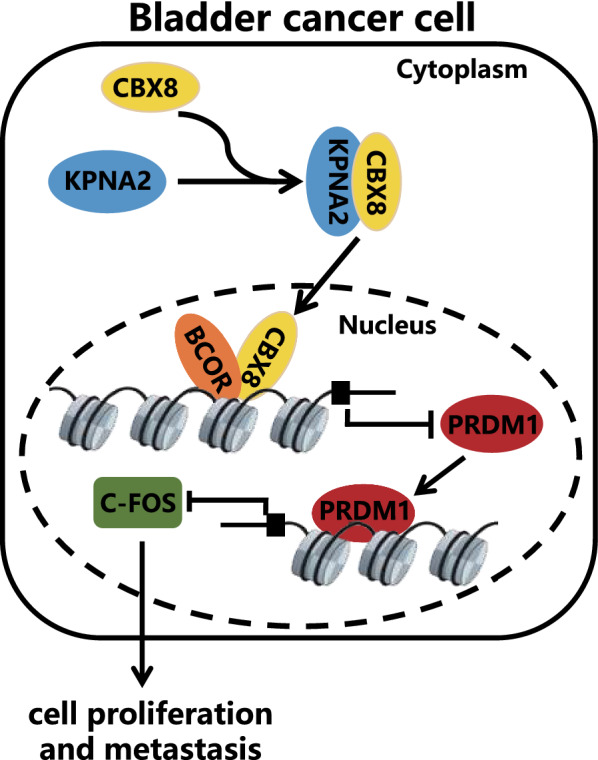
Graphic abstract showing how the KPNA2/CBX8/PRDM1/c-FOS pathway works in BCa. The interaction of KPNA2 with CBX8 contributes to the development and progression of BCa by mediating the PRDM1/c-FOS pathway

Collectively, our results allowed the decipherment of a functional axis whereby KPNA2 assisted the nuclear import of CBX8 to activate downstream effectors by recruiting BCOR to the promoter region of PRDM1 (see Fig. 7 for details), thus highlighting the tumor-promoting properties of KPNA2 in BCa. Our works hints at the possibility of using this axis to define a new therapeutic target to halt the development and progression of BCa.

## Data Availability

The datasets generated and analysed during the current study are available from the corresponding author on reasonable request.

## Abbreviations

ANOVA: Analysis of variance
BCa: Bladder cancer
CBX8: Chromobox 8
DEGs: Differentially expressed genes
DFS: Disease-free survival
ECM: Extracellular matrix
GAPDH: Glyceraldehyde-3-phosphate dehydrogenase
GEO: Gene Expression Omnibus
GEPIA: Gene Expression Profiling Interactive Analysis
KPN: karyopherin
KPNA2: KPN alpha 2
RT-qPCR: Reverse transcription quantitative polymerase chain reaction
PBS: Phosphate buffer saline
POD: Peroxidase
PPI: Protein–protein interaction
PRDM1: PR domain zinc finger protein 1
